# A novel splice variant of the stem cell marker LGR5/GPR49 is correlated with the risk of tumor-related death in soft-tissue sarcoma patients

**DOI:** 10.1186/1471-2407-11-429

**Published:** 2011-10-06

**Authors:** Swetlana Rot, Helge Taubert, Matthias Bache, Thomas Greither, Peter Würl, Alexander W Eckert, Johannes Schubert, Dirk Vordermark, Matthias Kappler

**Affiliations:** 1 Department of Radiotherapy, Martin-Luther-University Halle-Wittenberg, Halle(S), Germany; 2 Department of Oral and Maxillofacial Plastic Surgery, Martin-Luther-University Halle-Wittenberg, Halle(S), Germany; 3 Centre for Reproductive Medicine and Andrology, Martin-Luther-University Halle-Wittenberg, Halle(S), Germany; 4 Clinic of Urology, FA University Hospital Erlangen-Nürnberg, Erlangen, Germany; 5 Nikolaus-Fiebiger-Center for Molecular Medicine, FA University Erlangen-Nürnberg, Germany; 6 Department of General and Visceral Surgery, Diakoniekrankenhaus Halle, Halle, Germany

## Abstract

**Background:**

The human leucine-rich, repeat-containing G protein-coupled receptor (LGR) 5, also called GPR49, is a marker of stem cells in adult intestinal epithelium, stomach and hair follicles. LGR5/GPR49 is overexpressed in tumors of the colon, ovary and liver and in basal cell carcinomas. Moreover, an expression in skeletal muscle tissues was also detected. However, there has been no investigation regarding the expression and function of LGR5/GPR49 in soft-tissue sarcomas (STS) yet.

**Methods:**

Seventy-seven frozen tumor samples from adult STS patients were studied using quantitative real-time TaqMan™ PCR analysis. The mRNA levels of wild type *LGR5/GPR49 *and a newly identified splice variant of *LGR5/GPR49 *lacking exon 5 (that we called *GPR49Δ5*) were quantified.

**Results:**

A low mRNA expression level of *GPR49Δ5*, but not wild type *LGR5/GPR49*, was significantly correlated with a poor prognosis for the disease-associated survival of STS patients (RR = 2.6; P = 0.026; multivariate Cox's regression hazard analysis). Furthermore, a low mRNA expression level of *GPR49Δ5 *was associated with a shorter recurrence-free survival (P = 0.043). However, tumor onset in patients with a lower expression level of *GPR49Δ5 *mRNA occurred 7.5 years later (P = 0.04) than in patients with a higher tumor level of *GPR49Δ5 *mRNA.

**Conclusion:**

An attenuated mRNA level of the newly identified transcript variant *GPR49Δ5 *is a negative prognostic marker for disease-associated and recurrence-free survival in STS patients. Additionally, a lower *GPR49Δ5 *mRNA level is associated with a later age of tumor onset. A putative role of *GPR49Δ5 *expression in tumorigenesis and tumor progression of soft tissue sarcomas is suggested.

## Background

Treatment options for soft tissue sarcomas (STS) are often limited to surgery with the possibility of adjuvant chemotherapy and radiotherapy. The 5-year survival rate for STS patients is approximately 50% and depends strongly on the tumor stage [1]. Therefore, it is necessary to develop new prognostic markers with the potential to estimate the efficacy of an individual therapeutic strategy.

STS are a heterogeneous group of relatively aggressive tumors probably originating from adult mesenchymal stem cells (hMSCs) [2-4]. A study regarding a subgroup of STS (malignant fibrous histiocytoma; MFH) described hMSCs as the progenitors of MFH. Furthermore, the authors reported a novel tumor suppression role for Wnt signaling in solid tumors, which may have the potential for a new therapeutic strategy in sarcomas [5].

Here, we investigated the prognostic impact of the stem cell marker, cancer-associated gene and Wnt/Tcf4 target gene *LGR5/GPR49 *[6] in STS for the first time.

The leucine-rich repeat-containing G protein-coupled receptor LGR5/GPR49 has been identified as a novel stem cell marker in intestinal epithelia, stomach, and hair follicles [7-9]. Furthermore, *LGR5/GPR49 *mRNA was found to be expressed in normal human skeletal muscle tissues, which is of mesenchymal origin like STS [10].

*LGR5/GPR49 *mRNA is expressed in basal cell carcinoma (tumor of hair follicle), colorectal cancer and in tumors of the colon, ovary and liver [10-13]. Investigations of the *LGR5/GPR49 *protein expression are rare [14,15] because a full accepted antibody against LGR5/GPR49 is not available at the moment [6,16]. However, a prognostic impact of *LGR5/GPR49 *has not been shown so far.

The function of *LGR5/GPR49 *in tumorigenesis is supported by its ability to induce transformation of NIH3T3 cells in the presence of conditioned media from colorectal tumor cells. These findings indicate the possibility that the ligand of *LGR5/GPR49 *could be secreted by tumor cells [10]. McClanahan and colleagues therefore suggest a role for *LGR5/GPR49 *as a member of a novel class of transforming oncogenes and, as a result, a new potential molecular target for therapeutic intervention [10].

To our knowledge only one transcript variant of *LGR5/GPR49*, which lacks exon 8, is published in the ExPASy UniProtKB-data bank as variant VSP_037746 http://www.uniprot.org/uniprot/O75473. In this study, we identified a novel transcript variant of *LGR5/GPR49 *that lacks exon 5 (*GPR49Δ5*) (listed by us in the EMBL-Bank; http://www.ebi.ac.uk/ena/data/view/FN820440).

These variants are very interesting because both published *LGR5/GPR49 *variants have a truncated ligand binding extracellular domain [6,10]. It is possible that the affinity of these variants to the recently identified ligand of the full length *LGR5/GPR49 *gene product [17] is different or that different ligands could bind to the shortened receptor.

In summary, we report the first results for a newly identified variant of the *LGR5/GPR49 *gene, lacking exon 5, that we call *GPR49Δ5*. This is the first study that has demonstrated a prognostic impact of a LGR5/GPR49 variant in STS. In a multivariate Cox's regression analysis, we found that a low *GPR49Δ5 *mRNA level is an independent negative prognostic marker for disease-associated survival as well as for recurrence-free survival in STS patients.

## Methods

### Tissue samples and histopathological data

We examined frozen tumor samples from 77 STS patients using a real-time quantitative TaqMan™ analysis. The patients' median age was 59 years (ranging from 22 to 87 years). Forty-one patients (53%) died from their tumor after an average time of 28 months (ranging from 2-119 months), and 36 patients (47%) were still alive after an average observation period (i.e. after primary tumor resection) of 65 months (ranging from 11-146 months). The histopathological and clinical data has been summarized in Table 1 as described previously [18]. All tumor samples were collected before radio- or chemotherapy. After the tumor operation 27/77 patients were treated only with radiotherapy, two out of 77 patients were treated only with chemotherapy, and four patients were treated with a combination of chemotherapy and radiotherapy.

**Table 1:** Histopathological and clinical data

Category	*Patients*	*GPR49-mRNA level* *in zmol/molecule HPRT*		*GPR49Δ5-mRNA level in zmol/molecule HPRT*	
		≤1.07*10^-5^	> 1.07*10^-5^	≤7.6*10^-6^	> 7.6*10^-6^
*Total*	77	39	38	39	38
		P = 0.429		P = 0.212	
Men	35	16	19	15	20
Women	42	23	19	24	18
*Tumor stage*		P = 0.023*		P = 0.005*	
I	10	1	9	0	10
II	33	21	12	21	12
III	26	12	14	13	13
IV	8	5	3	5	3
*Tumor type*		P = 0.194		P = 0.166	
liposarcoma	19	6	13	5	14
fibrosarcoma/malignant fibrous histiocytoma	20	9	11	11	9
neurogenic sarcoma	6	3	3	3	3
rhabdomyosarcoma/leiomyosarcoma	22	15	7	14	8
Other STS	10	6	4	6	4
*Tumor resection*		P = 0.377		P = 0.172	
radical (R0)	51	24	27	23	28
not radical (R1)	26	15	11	16	10
*Patients at follow-up*					
alive	36	15	21	13	23
dead	41	24	17	26	15
*Localization*		P = 0.444		P = 0.207	
extremities	49	25	24	26	23
thorax	6	2	4	1	5
head	1	0	1	0	1
abdomen	19	10	9	10	9
multiple	2	2	0	2	0
*Metastasis*		P = 0.291		P = 0.025*	
*M0*	21	9	12	*7*	14
*M1*	29	17	12	*19*	10
*Age of tumor onset*		P = 0.218		P = 0.039*	
*age (mean)*		59.9	55.4	61.4	53.9
*Kaplan-Meier analysis*		P = 0.056		P = 0.004*	
mean survival (months)		50.1 ± 6.5	80.3 ± 10.7	45.8 ± 6.4	86.2 ± 10.9
*Univariate Cox's Regression*		P = 0.06		P = 0.006*	
RR		1.8		2.5	
CI		0.97-3.5		1.3-4.8	
*Multiv. Cox's-Regression*		P = 0.12		P = 0.026*	
RR		1.8		2.6	
CI		0.9-4.0		1.1-6.0	

The study was carried out in compliance with the Helsinki Declaration, and it was approved by the Ethics Committee of the Medical Faculty of the University Halle. All patients gave written informed consent (Department of Surgery 1, University of Leipzig, Germany).

### Quantitative RT-PCR

Isolation of total RNA and cDNA synthesis were performed according to standard protocols as described previously [19]. The cDNA was amplified by automated real-time quantitative TaqMan™ assays for the wild type LGR5/GPR49 and GPR49Δ5 transcript variants and Hypoxanthin- guanine -phosphoribosyltransferase (HPRT) transcript using kits from Roboscreen (AJRoboscreen GmbH, Leipzig, Germany).

Wild type *LGR5/GPR49 *and *GPR49Δ5 *transcript amounts were correlated to *HPRT *transcript amounts as zeptomole (zmol, 10^-21^) wild type *LGR5/GPR49 *or *GPR49Δ5 *mRNAs per molecule *HPRT *mRNA in duplicate measurements.

An elevated expression of wild type *LGR5/GPR49 *was determined as an expression of > 1.07 × 10^-5 ^zmol wild type *LGR5/GPR49 *mRNA/molecule *HPRT *mRNA and for *GPR49Δ5 *as an expression of > 7.6 × 10^-6 ^zmol *GPR49Δ5 *mRNA/molecule *HPRT *mRNA.

### Statistical analysis

Cox's regression hazard models and Kaplan-Meier analyses were used to estimate a correlation of wild type *LGR5/GPR49 *and *GPR49Δ5 *mRNA with disease-associated survival of STS patients. Cox's regression hazard model was adjusted to the prognostic effect of covariates (gender of patients, tumor stage, tumor entity, tumor localization, and type of tumor resection), and the relative risk (RR) was calculated. For analyzing the recurrence-free survival of STS patients, a Kaplan-Meier analysis was performed. The follow-up time starts with the day of primary tumor operation. The end point for the disease-associated survival analysis was the time of death of the patient. The end point for the recurrence-free survival analysis was the first recurrence. The interrelationship between gene expression levels was tested with the Spearman's rank correlation (r, correlation coefficient). A probability (P) of < 0.05 was defined as significant. Statistical analyses were carried out using SPSS software version 17.0 (SPSS Inc., Chicago, USA).

## Results

### Correlation of the mRNA expression level with survival of the STS patients

The median transcript ratios of 77 STS samples were 1.07 × 10^-5 ^(ranging from 0-4.1 × 10^-3^; mean 2.3 × 10^-4^) zmol wild type *LGR5/GPR49 *mRNA and 7.6 × 10^-6 ^(ranging from 0-6.4 × 10^-3^; mean 2.5 × 10^-4^) zmol *GPR49Δ5 *mRNA/molecule *HPRT *mRNA, respectively.

For the survival analysis, STS patients' data were separated according to the median expression levels. GPR49 low expression was defined as ≤1.07*10^-5 ^GPR49 mRNA/molecule HPRT mRNA (39 patients) and high expression was defined as > 1.07*10^-5 ^GPR49 mRNA/molecule HPRT (38 patients). For GPR49Δ5, low expression was defined as ≤7.6*10^-6 ^GPR49Δ5 mRNA/molecule HPRT mRNA (39 patients and high expression was defined as > 7.6*10^-6 ^GPR49Δ5 mRNA/molecule HPRT) (38 patients). The separation was based on the median expression level of the wild type LGR5/GPR49 mRNA and GPR49Δ5 mRNA (see also Table 1).

**Figure 1:**
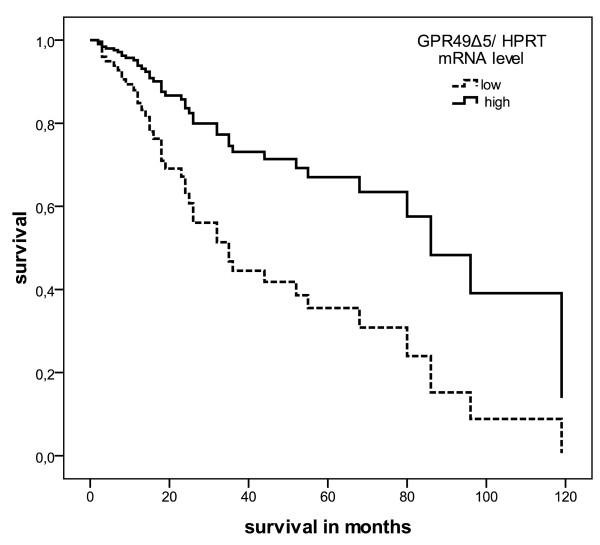
**Multivariate Cox's hazard regression model for *GPR49Δ5 *mRNA expression level and disease-associated survival in STS patients**. Expression level of *GPR49Δ5 *mRNA in STS of 77 patients was correlated with disease-associated survival. The model was adjusted to patients' gender, tumor stage, tumor localization, tumor entity, and the type of tumor resection. The high and low cut-off values for *GPR49Δ5 *were > 7.6 × 10^-5 ^and ≤7.6 × 10^-5 ^zmol *GPR49Δ5 *mRNA/molecule *HPRT *mRNA (RR = 2.6, P = 0.026).

We performed a Kaplan-Meier analysis, which detected that STS patients with a lower intratumoral expression of wild type *LGR5/GPR49 *mRNA died an average of 30 months earlier (P = 0.056), with lower intratumoral *GPR49Δ5 *mRNA expression died an average of 40 months earlier (P = 0.004) than patients who had higher intratumoral expression of wild type *LGR5/GPR49 *mRNA and *GPR49Δ5 *mRNA, respectively (Table 1). Multivariate Cox's regression hazard analysis revealed an increased risk (RR = 1.8, P = 0.12, Table 1) of tumor-related death for patients with a low wild type *LGR5/GPR49 *mRNA level and a 2.6-fold (P = 0.026) increased risk for STS patients with a low level of *GPR49Δ5 *mRNA in their tumors, respectively (Figure 1).

**Figure 2:**
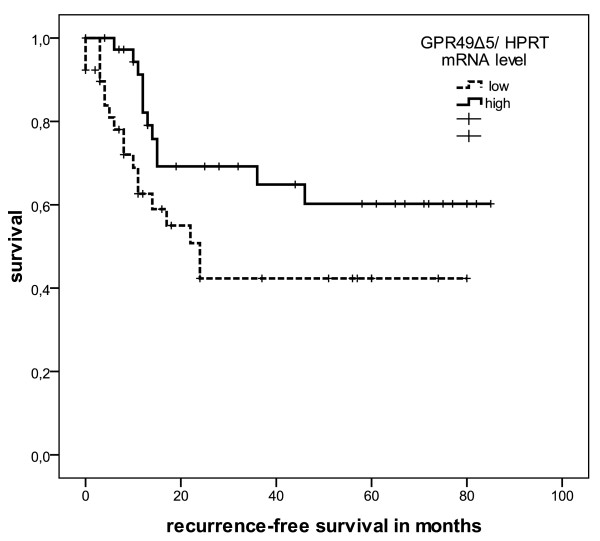
**Kaplan-Meier analysis recurrence-free survival curves according to *GPR49Δ5 *transcript levels**. Expression of *GPR49Δ5 *mRNA for 77 patients with STS was correlated with recurrence-free survival. The high and low cut-off values for *GPR49Δ5 *were > 7.6 × 10^-5 ^and ≤7.6 × 10^-5 ^zmol *GPR49Δ5 *mRNA/molecule *HPRT *mRNA (P = 0.043).

Moreover, when we estimated recurrence-free survival, a low level of *GPR49Δ5 *mRNA expression was significantly associated with an early recurrence (P = 0.043; Figure 2), whereas low wild type *LGR5/GPR49 *mRNA expression levels had no significant effect (P = 0.20).

**Figure 3:**
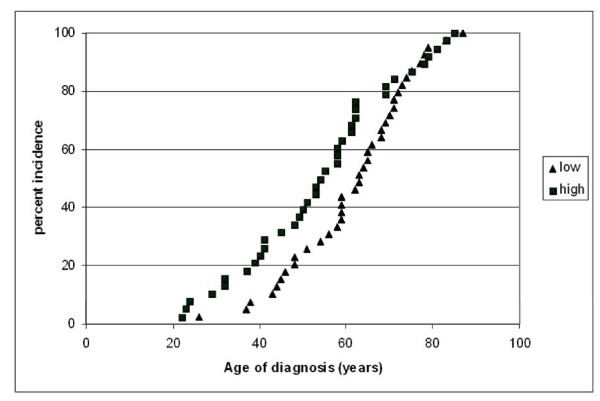
**Correlation of *GPR49Δ5 *mRNA expression with age of tumor onset**. There is a significant difference in the onset of the tumor (7.5 yrs) between STS patients with a low level (> 7.6 × 10^-5 ^zmol *GPR49Δ5 *mRNA/molecule *HPRT *mRNA) (61.4 yrs) compared to patients with a high level (≤7.6 × 10^-5 ^zmol *GPR49Δ5 *mRNA/molecule *HPRT *mRNA) of *GPR49Δ5 *tumor mRNA expression (53.9 yrs) (P = 0.039).

Furthermore, we found a significant difference in tumor onset (7.5 yrs) between STS patients with a low level compared to patients with a high level of *GPR49Δ5 *mRNA expression (61.4 vs. 53.9 yrs) in their tumors (P = 0.039; Student's t-test, Table 1; Figure 3).

### Correlation with clinical parameters

Bivariate analysis demonstrated that low expression of wild type *LGR5/GPR49 *mRNA correlated with higher tumor stage (P = 0.02). Low expression of *GPR49Δ5 *mRNA was also significantly associated with higher tumor stage (P = 0.005) and, moreover, with the occurrence of late distant metastases (P = 0.025) (Table 1). The mean intratumoral expression of *GPR49Δ5 *mRNA in the primary tumor was a 6fold lesser (6.9 × 10^-5 ^vs. 4.3 × 10^-4 ^zmol *GPR49Δ5 *mRNA/molecule *HPRT *mRNA) for those patients (n = 29) who developed metastases compared to patients (n = 21) without metastases.

## Discussion

In this study, we demonstrated that a low *GPR49Δ5 *mRNA level is a negative prognostic marker for disease-associated and recurrence-free survival in STS patients (Table 1).

LGR5/GPR49 labels stem cells in intestines, stomach and hair follicles [7-9]. When compared to corresponding normal tissues, *LGR5/GPR49 *mRNA is overexpressed in human hepatocellular carcinomas, basal cell carcinoma, colon-, colorectal- and ovarian tumors [10-13,20]. However, Yamamoto *et al*. found no significant association between expression of LGR5/GPR49 and clinicopathologic features of tumors [12]. We found that low mRNA expression of *GPR49Δ5 *has a prognostic impact for STS patients.

Why does low expression impact survival? We found that low expression of wild type *LGR5/GPR49 *mRNA (P = 0.023), as well as low expression of *GPR49Δ5 *mRNA (P = 0.005), was significantly associated with a higher tumor stage. STS patients with high-stage tumors have a poorer prognosis than those with low-stage tumors [21]. In accordance with our results, McClanahan and colleagues observed that in both colorectal and ovarian carcinomas expression of *LGR5/GPR49 *mRNA was high in stage I and II tumors and appears to decrease in stage III and IV tumors. The authors suggest that overexpression of *LGR5/GPR49 *may be an early event in tumorigenesis [10]. Furthermore, using a mouse endometrial cancer model, Sun *et al*. found that *LGR5/GPR49 *mRNA is highly expressed in the epithelium during the initial stages of tumorigenesis, but is remarkably down-regulated in full-grown tumors [22]. Fan *et al*. postulated a function for LGR5/GPR49 in the initiation of colorectal carcinomas [14]. This hypothesis corresponds with our finding that patients with lower-staged tumors had higher *GPR49Δ5 *mRNA levels, but earlier tumor onset.

However, low expression of *GPR49Δ5 *mRNA appears to be associated with tumor progression. Low expression of *GPR49Δ5 *mRNA is correlated with poor recurrence-free survival (P = 0.043) in STS patients and with the occurrence of late distant metastases (P = 0.025). Uchida *et al*. described diffuse *LGR5/GPR49 *mRNA expression in the entire tumor, as well as in the invasive front of human colorectal carcinomas [20]. *LGR5/GPR49 *mRNA expression in colorectal carcinomas correlated significantly with the number of lymph node metastases, lymphatic invasion, and vascular invasion [20]. Moreover, the expression of LGR5/GPR49 mRNA was significantly associated with poor prognosis for disease-free survival of colorectal cancer patients [11]. These data suggest, that *LGR5/GPR49 *mRNA expression may be associated with the malignant potential of colorectal cancer, including the appearance of metastasis [20].

Recently and in accordance to our results, Walker and colleagues described that an ablation of LGR5 induces increased invasion and anchorage-independent growth, and enhances tumourigenicity in xenografts experiments of human colorectal cancers. Suppression of LGR5/GPR49 leads to a strong upregulation of mesenchymal genes and of genes positively regulating EMT (Epithelial to Mesenchymal Transition) - the same genes are markedly downregulated upon LGR5 overexpression [23]. These findings suggest that LGR5/GPR49 is important in restricting stem cells to their niche, and that loss of LGR5 concomitant with activated wnt signalling may contribute to the invasive phenotype of colorectal carcinomas. These results highlight the importance of LGR5, not simply as marker of colorectal tumor cells, but as a regulator of wnt responses, cell motility and cell-cell adhesion [23].

This fact can be of importance even for tumors of mesenchymal origin like STS.

## Conclusion

In this study, we analyzed mRNA expression of an additional stem cell-associated gene, *LGR5/GPR49 *and its splice variant *GPR49Δ5*, for its association with disease-associated and recurrence-free survival. We were able to identify *GPR49Δ5 *mRNA expression as an independent prognostic factor for STS patients. We suggest that stem-cell-associated genes play a major role in sarcoma patients' prognosis and, therefore, could be valuable targets for future therapy. Together, our results suggest a role of GPR49Δ5 in tumorigenesis and tumor progression of STS.

## List of abbreviations

GPR49: G protein-coupled receptor; MFH: malignant fibrous histiocytoma; hMSC: human mesenchymal stem cell; HPRT: hypoxanthine phosphoribosyltransferase; LGR5: leucine-rich repeat-containing G protein-coupled receptor; r: correlation coefficient (spearman test); RR: relative risk; STS: soft tissue sarcomas; Tcf4: T-cell transcription factor 4.

## Competing interests

The authors declare that they have no competing interests.

## Authors' contributions

SR, HT, DV and MK designed the study, collected data, performed statistical analysis and drafted the manuscript. PW treated the patients, collected material and data and reviewed the manuscript. MB and TG made substantial contributions to the acquisition and interpretation of data. AE and JS were involved in drafting the manuscript and revising it critically. All authors read and approved the final manuscript.

## Pre-publication history

The pre-publication history for this paper can be accessed here:

http://www.biomedcentral.com/1471-2407/11/429/prepub
